# Indents

**Published:** 1922-12

**Authors:** 


### INDENTS

**☞Brief Articles of Technical or Scientific Value will receive notice and comment. Contributions invited.**

---

CONSERVING PUBLIC HEALTH. It is of interest to note how a slow, but gradual interest in conservation of the teeth and healthful conditions of the mouth is growing throughout the nation. Already a few states have enacted state laws with financial aid, towards a state-wide teaching and professional care of the children, in regard to their mouths. If we are to take our place among those intense in their desire to decrease the ravages of ill breath and to build up from infancy a better and stronger race, then we should constantly be on the alert to give our aid and our support to any and all measures of this nature. As time goes on there will be put in operation, in various places, an organized effort to try out by interesting ways and means, methods to study conditions leading towards better breath of our youth. Dentists in such locations, which may be chosen, will certainly fall short of their opportunity, should they neglect to insist that part of such work should carry in its scope mouth conditions. The old adage that "the Lord helps those who help themselves" is still true. Also, we must keep continually urging in our many localities the fact that the thousands of wards of the state must receive adequate dental service. Not a single member of the legislature has his own home dentist, and it should be, it must be, the prerogative of such dentist to educate his legislative friend and patient to the deplorable condition prevailing in many of our institutions. I am glad to say that within the past few years a few institutions are doing the best they can with limited means to give dental service, and that the State Board of Administration, with its financial restrictions, are recognizing the needs of dental service. Especially should the desirability of the health of the mouth and the conservation of the teeth of our school and pre-school youth be kept before our school authorities. —H. M. Semans, in *Summary*.

---

Injudicious Extraction of Teeth. The injudicious extraction of teeth in the acute stage of abscess has been a bone of contention for a great many years, and I feel this should be thrashed out to the satisfaction of all at the earliest possible time.

When such a case presents itself, the history and peculiarities of the individual case never seem to enter into consideration with some men; the only thing they see is a swollen jaw, a loose tooth, and, if they are observing enough, an ill-appearing patient. Never do they stop to consider what causes the illness of the patient; what causes the rise of temperature; what causes the loss of appetite, the restlessness and *malaise* of the patient. Upon examination it will be found that the soft tissues are baldly indurated and hot, with no evidence of localization of pus, but a diffuse infection extending well into the parotid, submaxillary and sublingual regions. When such a condition exists, extraction is contra-indicated and should be advised against; palliative treatment should be given and continued until there is a definite localization of the pus, when this should be drained.

If the case is allowed to rest until the tissues have regained their normal condition, save for the drainage of the pus, less post-operative treatment will be necessary and less reaction will result from the operation. If, however, the tooth is extracted during the acute stage, with conditions existing as stated above, a cellulitis is very apt to occur followed by osteomyelitis and necrosis, if not a Ludwig's angina following the cellulitis; in such a case prognosis is not favorable. Many cases of general septicemia have been a direct result of the extraction of a tooth under conditions as described above. These cases should undoubtedly be placed in the hands of a competent oral surgeon and handled in a most serious manner. —A. L. Miller, in *Cosmos*.

---

EXTRACTING INFECTED TEETH. Hubeny in a recent paper gives the medical viewpoint quite concisely when he says that we as dentists are assuming a greater responsibility than we are justified in doing when we attempt by an embalming process to retain or restore an organ manifestly dead and septic. The fault, he contends, is palpably the patient's, since it is lack of care on his part that has brought him to this pass, so why, he asks, should we be keen to accept a burden fraught with such dangerous possibilities?

In tooth extraction, as a matter of course, we invariably have the patient on the side of conservation, unless he is ill enough to be desperate, but this should receive as little consideration from us as would a patient's disinclination to undergo a needed major operation from the surgeon. If the patient's health, present or future, demands extraction, *let it be done.*

There are always two factors to be considered in every case of existent or potential metastatic pathologic invasion: the virulence of the infection and the resistance of the patient. So long as resistance is uppermost, of course, vital balance is maintained. Neither of these factors, however, may be predetermined, nor can we say in how short a space of time the balance may be overcome. Therefore, when we assume to dictate whether or not manifestly septic conditions should be ignored for tooth conservation, we are going beyond our field. This decision is undeniably the prerogative of the physician and we should wherever possible defer to him utterly. Such co-operation and collaboration is desirable and essential.

The physician on the other hand must look to the dentist for oral diagnosis and the dentist should be prepared to furnish him with definite knowledge as to conditions. This presupposes his ability to do so—a supposition, unfortunately, not always justified by fact.

A diagnosis of oral conditions must be based primarily on an interpretable radiogram, a complete oral radiogram if possible in every case. The desirable radiogram is one produced without over- or under-exposure, properly developed, and without distortion. If any confusion arises over any area shown, this should be re-exposed to provide multiple views of the teeth or edentulous area in question. —F. F. Molt, in *Summary*.

---

Teeth and Health. To extract useful teeth that can and should be saved is an injustice to the public that should be and is becoming severely criticised. On the other hand, the effect of diseased teeth upon general health is so far-reaching that much emphasis should be placed upon the importance of removing or correcting such conditions. The dentist is placed in a very difficult position and must make a very careful diagnosis of each case. To avoid pulp removal where healthy conditions exist is a most important consideration but there are cases of hyperemia and inflammation or acidental exposure constantly presenting for treatment which must be taken care of. Useful teeth should not be sacrificed when it becomes necessary to remove the pulp. These teeth require pulp treatment and canal filling and this service should be given in such a way as to prevent future periapical disturbance. Frequently the requirements of large artificial restorations and crowns or attachments for bridges and partial dentures are so severe that a hypersensitive condition results, or even the death of the pulp. Many such cases would be less disastrous to the patient's health and much more comfortable if the pulp had been removed and the root canal properly filled before such construction was begun.

Dentistry has been dealing with teeth rather than with health. The field of dentistry today is no longer confined to the realm of teeth, nor even to the mouth, for there is an intimate relation between the teeth, the gingivae, the periapical tissue and the general health of the individual. In determining the procedure in every case the question must be considered, "what relation has this operation to the health and comfort of the patient." The same recommendation can not be given in all cases and no set rule can be made for determining what teeth should be retained and what ones eliminated. The general health of the patient must always be a basis from which the plan of operation is constructed. More exacting treatment should be given where vitality has been lowered and general health affected by absorption of infectious and toxic products. The condition of the tissue surrounding the tooth itself is the next of importance. Where diseased conditions of this tissue can be corrected by treatment and eliminated as a source of infection such teeth should be saved. The easier method of extraction should be condemned. The age of the patient is an important factor in the diagnosis. There are greater chances of a response to treatment in the young patient that in more advanced ages. Since the reconstruction of the masticating organs so that they can function properly is so important in its relation to health there should be a very careful consideration of the needs of these requirements in the examination and plan of operation in each case. The loss of molar teeth, and especially all molars on one side of the arch, is far more serious than many seem to consider it. To cause a young patient to go through life with no molar teeth or with an artificial denture may do more harm to the health of the patient than would the teeth which were unwisely extracted.—E. D. Coolidge, in *Summary.*

THE INCREASING OCCURRENCE OF GANGRENOUS ULCERATIONS. Plaut's observations are confirmed. There is an increased occurrence of these affections in Berlin also. The fuso-spirillary organisms were always present. They are, however, not to be held primarily responsible, for it is believed that some invisible, unknown virus must be the cause. As with ulcus molle, no auto-infection occurs in these cases.—By A. Buschke.

---

ALCOHOL AS SURGICAL DISINFECTANT. Cignozzi asserts that addition of 70 per cent. of ethyl alcohol or of 35 per cent. propyl alcohol modifies the surface tension of disinfectants and renders them much more penetrating and bactericidal. The formula found most effectual in his long experience has been 70 per cent. ethyl alcohol containing 1 or 0.5 per cent. acetic acid.—By O. Cignozzi, *Cosmos*.

